# A Novel Application of an Anthelmintic Mixture for Use against Gastrointestinal Parasites of Red Deer* (Cervus elaphus)*

**DOI:** 10.1155/2018/6024920

**Published:** 2018-03-05

**Authors:** P. L. Hughes

**Affiliations:** Taihape Veterinary Services, Kotare Street, Taihape 4720, New Zealand

## Abstract

A mixture of proprietary anthelmintics delivering 0.5 mg/kg moxidectin, 9.06 mg/kg oxfendazole, 15 mg/kg levamisole, and 0.08 mg/kg selenium on bodyweight basis per os to red deer is investigated. On a deer farm with a history of parasite problems, six weaner red deer were treated orally with a 50/50 mixture of Exodus Pour-On and Oxfen C Plus (Ex/Ox) at a dose rate of 1 ml/5 kg bodyweight. Six herd mates were untreated. Eleven days later abomasal worm counts for the untreated deer revealed an arithmetic mean burden of 2,566* Ostertagia*-type worms and 300* Trichostrongylus axei*. No worms were detected in the abomasa of the treated group. Six yearling red deer were treated with the Ex/Ox combination and sent 39 days later to a slaughter plant where tissue samples were collected for residue analysis. Moxidectin was the only anthelmintic compound to show residues and the concentrations measured were well below maximum residue limits. Laboratory analysis of the Ex/Ox product after six-week storage at ambient temperature indicated good physical and chemical stability. These investigations support the hypothesis that the Ex/Ox combination can be an effective and practical anthelmintic option for use in red deer against a background of widespread gastrointestinal parasite resistance to the registered alternatives.

## 1. Introduction

It has been widely recognised by farmers and veterinarians in the deer industry for a number of years that the efficacy of anthelmintics used has decreased especially against* Ostertagia*-type strongyles [1, 2]. Moxidectin pour-on, which until recently was the most common anthelmintic used on deer farms [3], has become ineffective to the point where it is now recommended that its use be discontinued [4]. Deer farmers in the Taihape district are generally aware of this problem and have largely ceased using the macrocyclic lactone (ML) pour-ons despite their obvious convenience over both oral and injection methods of treatment.

Moxidectin, however, still offers a considerable advantage against lungworm* Dictyocaulus eckerti* in deer [5]. In the Taihape region there has been a noticeable increase in lungworm disease in weaner deer where abamectin or eprinomectin has been used instead of moxidectin even when used in combination products, for example, Matrix C (Merial Ancare, Auckland, NZ). There has not been a recorded case of reduced efficacy of moxidectin against lungworm in deer, so persistent activity would still be expected to be maintained for 35–42 days against this parasite [5].

A combination product including moxidectin is therefore desirable. The pour-on formulations are not now helpful, leaving injectable and oral formulations as possible alternatives. There are studies comparing the efficacy and plasma pharmacologic profiles of these two alternatives in deer [4, 6, 7]. On balance it would appear that the injectable formulation has higher efficacy but comparisons do show variability. Furthermore the dose rates used are extrapolated from other farmed ruminants species and the oral dose rate for moxidectin has not been established for deer.

Oral formulations are preferred by farmers in the Taihape region because of the pain expressed by deer after repeated skin injections and some needle injuries. Furthermore anthelmintic treatment has traditionally been achieved with a single application and farmers tolerate a considerable cost premium to maintain that convenience. The challenge then was to provide a highly concentrated mixture of the three active compounds so that a practical oral dose volume was achieved.

To achieve this goal a mixture of proprietary anthelmintics (Ex/Ox) was proposed. The concern of administering a pour-on formulation per os was tempered by the fact that manufacturers recognise that pour-on formulations can be and are ingested via licking the application site. Exodus Pour-On contains 5 mg/ml moxidectin suspended in a soya bean oil and benzyl alcohol base neither of which would be expected to be toxic at the dose rates proposed to mammals (Merial Ancare Exodus Pour-On Safety Data Sheet). The first animals to receive the Ex/Ox combination treatment were the authors own deer (in mid 2013) and no ill effects were recorded. The dose administered was derived from aiming to at least double the quantities of the three active compounds in a practical volume, with 1 ml/5 kg bodyweight being commonly used for oral drenches. A combination of 0.5 mg/kg moxidectin, 9.06 mg/kg oxfendazole, 15 mg/kg levamisole HCI, and 0.08 mg/kg selenium was administered. This dose provides moxidectin at 2.5 times the “standard dose rate” and oxfendazole, levamisole, and selenium at twice the “standard dose rate” based on their use in sheep and cattle. The quantity of selenium included in this combination while a potential cause of toxicity was considered but regarded as not critical as previous work has shown that young deer are not particularly sensitive to an increase in selenium administration [8]. The most toxic anthelmintic compound in this combination for ruminants is levamisole [9].

The purpose of this investigation was to provide clinical information on the Ex/Ox drench combination which is now in common use in the Taihape region. The data provided here may enable veterinarians to make a more informed decision should they prescribe this anthelmintic combination to their deer farmer clients. The observations relate specifically to the use of this mixture of commercial anthelmintics and are confined at this stage for use in red deer. Wapiti and wapiti hybrid deer have been shown to be more susceptible to G/I parasites [10] so care is required in making extrapolations with those breeds.

## 2. Materials and Methods

The anthelmintic combination comprised Exodus Pour-On (Merial NZ Ltd, Auckland, NZ) and Oxfen C Plus (Merial NZ Ltd, Auckland, NZ). Exodus Pour-On has a label claim of 5 mg/mL moxidectin in a benzyl alcohol/soya bean oil solution. Oxfen C Plus has a label claim of 90.6 mg/mL oxfendazole, 150 mg/mL levamisole, and 0.8 mg/mL selenium in an aqueous suspension/solution with approximately 12% w/v of stabilisers, emulsifiers, and suspending agents. Each anthelmintic was mixed in equal amounts in a 5 L measuring cylinder. The mixture was poured into a 5 L drench pack and shaken to form a thick viscous solution which could pass through a normal oral drench gun system.

There were three components to the clinical investigation into the use of this anthelmintic mixture in red deer.

### 2.1. Efficacy

A 730 hind breeding and finishing property with a parasite problem in deer had already used the Ex/Ox combination in their deer. Most deer farms in the Taihape region are in this category. Faecal worm egg counts (FEC) from a mob of weaner deer provided an indication as to whether a sufficient gastrointestinal (G/I) worm burden was established to warrant further treatment and were made by the modified McMaster method [11]. These deer had been drenched 3.5 months earlier using the Ex/Ox combination when still with their dams. Six weaner mixed sex red deer were weighed and treated. Six undrenched deer of similar weight provided an on-farm control group. A new drench gun was used to give dose to individual bodyweight. The drench gun had been calibrated by checking the volume administered with a syringe at the start and twice more during administration to ensure accuracy. The tag numbers, weights for both groups, and volume of Ex/Ox combination used on the treated deer are presented in Table 1.

Eleven days after drenching (27th June 2016) all 12 deer were tranquilised and humanely slaughtered on farm and post mortem examination of the viscera was carried out. The lungs were all thoroughly examined to detect lungworm. Livers, abomasa, and small intestines were individually identified and sent to Gribbles Palmerston North Laboratory. Worm counts were carried out on the individual abomasa and small intestinal samples which were recorded against the tag number. These worm counts were based on examinations of 1% aliquots of abomasal and small intestinal washings and the contents passed through a 250 *μ*m mesh sieve. This procedure has been shown to reliably detect all but the early 4th stage larvae [12]. Species composition of worm genera was based on examination of the spicule morphology of 50 randomly selected male worms recovered from pooled samples made up of equal-sized aliquots from all animals in the control group.

The liver samples were individually analysed for selenium concentrations.

### 2.2. Residue

Six rising one-year-old mixed sex red deer were individually identified by tag number, weighed, and drenched with 23 ml of the Ex/Ox combination on the 20/8/15. The average weight of the six deer was 99 kg, range 87–115 kg. The dose rate of 23 ml was chosen because it represented the dose rate applicable to the heaviest animal which is an industry recommendation. These six deer were sent with a line of 40 undrenched herd mates to a deer slaughter plant (DSP) by the author. Full documentation for all the deer was provided to the DSP manager and veterinarian. The date of slaughter was 28/9/15, 39 days after treatment. The DSP veterinarian collected muscle, liver, kidney, and fat samples from the six treated deer and from two further deer from the undrenched cohorts. The six treatment carcasses were discarded. All 32 tissue samples were sent to RJ Hills Laboratories, Hamilton. At this laboratory the samples were homogenised to a pulp with dry ice. A subsample of the homogenised sample was extracted with a sodium carbonate solution and acetonitrile. Salt was added to the extracts and shaken to separate the acetonitrile from the aqueous phase. An aliquot of the acetonitrile extract was cleaned up by dispersive Solid Phase Extraction, diluted, and then analysed by liquid chromatography mass spectroscopy for fenbendazole, fenbendazole sulfone, levamisole, moxidectin, and oxfendazole.

### 2.3. Stability

Samples of the Ex/Ox anthelmintic combination were made using newly made commercial products and stored in high-density polyethylene plastic containers both at room temperature and at 2–8°C for 6 weeks. After storage the samples were shaken vigorously as per normal farm practice and the appearance of the mixture and its density were evaluated and the moxidectin, oxfendazole, and levamisole HCl contents were measured. This was carried out at Labtec Scientific and Technical Services, an International Accreditation New Zealand accredited independent laboratory. Moxidectin, oxfendazole, and levamisole HCL were all extracted in acetonitrile and were separated by reverse phase high performance liquid chromatography. Detection was by ultraviolet light absorption at 242 nm for moxidectin and 210 nm for oxfendazole and levamisole HCL. Quantitation was by external standardisation.

## 3. Results

### 3.1. Efficacy

Arithmetic mean pretrial FEC of 280 strongyle eggs/g indicated parasites were present in the mob under investigation.

The abomasal worm counts for the treatment group and the untreated group are presented in Table 2.

No parasites were found in the small intestine of any of the untreated group and no signs of lungworm infection were found in any of the deer at post mortem. Speciation of the* Ostertagia*-types revealed that 90% were* Spiculopteragia asymmetrica*, 6%* Spiculopteragia spiculoptera,* and 4%* Ostertagia leptospicularis*.

Analytical results of selenium content in the livers are shown in Table 3.

### 3.2. Residue

Residue concentrations for all samples were below the default detection limit (0.005 mg/kg) for fenbendazole, fenbendazole sulfone, oxfendazole, and levamisole. Residues in all kidney and liver samples were below the default detection limit for moxidectin. The residue concentrations for moxidectin in fat and muscle are shown in Table 4.

### 3.3. Stability

The Ex/Ox mixture showed good physical and chemical stability over the 6-week storage period at both 2–8°C and room temperature. It maintained its appearance as a white suspension, with no change in density (1.022 g/mL) over that time. Assay results after 6 weeks of storage showed concentrations of 2.7, 45.5, and 78.9 mg/mL for moxidectin, oxfendazole, and levamisole, respectively. Based on the label claims and 50% dilution of each product the expected assay concentrations would be 2.5, 45.3, and 75 mg/mL, respectively.

## 4. Discussion

Elimination of abomasal parasites was achieved with the oral dose of 1 ml/5 kg bodyweight of the Ex/Ox combination, including a large burden of* Ostertagia*-type parasites with three different species represented. Investigations by Lawrence 2011, 2012, and 2013 have confirmed that* Spiculopteragia asymmetrica*,* Spiculopteragia spiculoptera*, and* Ostertagia leptospicularis* have become resistant to moxidectin pour-on treatment. All three species were represented in the worm burdens of the animals on the farm in Taihape where this investigation was conducted.

*Spiculopteragia asymmetrica* was the most common species identified on this farm. Interestingly in another study by Lawrence et al. [6], moxidectin at a dose rate of 0.2 mg/kg per os only achieved a 91.8% efficacy against this parasite. This study was on a Te Anau property that had recognised a poor clinical response to moxidectin pour-on, similar to that on the Taihape property. Greater than 99.9% efficacy was shown for this parasite in the Taihape case, which is encouraging and suggests that a 0.5 mg/kg dose rate of moxidectin orally may be more appropriate at least in this combination mixture.

The anthelmintic resistance status of the Taihape property is unknown but it has a similar history to many others in the Taihape district, namely, more than 20 years of regular moxidectin and abamectin pour-on use. Although there was no specific diagnosis of ML resistance, there was sufficient evidence and concern to move to the “off label” Ex/Ox combination drench. Despite earlier work [5, 13] showing high efficacy for moxidectin pour-on, the observations of farmers and veterinarians in this district is that there are likely now very few deer farms where moxidectin pour-on is efficacious against the* Ostertagia*-type parasites.

A problem for veterinarians in the deer industry is that the faecal egg count reduction test (FECRT) is not as helpful as it is in sheep for example. Previous studies by Anderson and Wilson [14] have shown that there is a poor correlation between FEC and total worm burdens in deer. In addition, studies by Mackintosh et al. [7] have shown that the FECRT in deer overestimates the efficacy of anthelmintics and is therefore an unreliable diagnostic test. Hence the FECRT cannot be used to establish ML resistance status of a property and while we had collected faeces from farms using the Ex/Ox combination drench showing zero FEC 10–14 days after drenching this finding cannot be used to support high drench efficacy. This was the reason that the postslaughter investigation was undertaken on the farm in Taihape.

Selenium liver analysis was carried out to determine what the changes in concentrations were after dosing at 0.2 mg/kg sodium selenate. The concentrations were below the adequate range in the untreated group and above in the treated group. This was surprising because these weaner deer had been treated 3.5 months prior on their dams with the Ex/Ox combination. This finding illustrates that selenium can be quite rapidly depleted in deer and on this property something in the order of 6 weekly dosing at the higher dose rate of Ex/Ox could be required to maintain liver concentrations in the adequate range. It does need to be stated however that selenium responsive conditions in deer have been reported rarely [15]. Toxicity is the concern and while toxic reference ranges have not been published for deer, toxic concentrations in cattle livers have been above 80,000 nmol/kg [16]. It would seem highly unlikely that concentrations of that magnitude would be breached by using the Ex/Ox combination even if overdosing occurred (e.g., at double rates) and it was used monthly. The safety of the Ex/Ox combination is supported by its use in several thousand weaner deer over a four-year period. A six-week dosing interval for rising one-year-old deer from weaning until spring pasture flush (3–5 drenches total) appears to work well in many commercial situations.

Moxidectin was the only anthelmintic compound to show residues, an expected finding given the pharmacology of the three actives, and the WHP of 10 days for levamisole and benzimidazoles in deer. It was expected that levamisole and the benzimidazole would be metabolised within 35 days and that moxidectin would persist in low levels in fat (J Hurst pers. com.). Five of the 6 treated and one of the controls had moxidectin residues in fat. The highest was 0.070 mg/kg and mean was 0.033 mg/kg. The Ministry of Primary Industry (MPI) published maximum residue limit (MRL) for moxidectin in fat of deer is 0.5 mg/kg [17]. The mean residue was 6.5% of the MRL. Two muscle samples had moxidectin residues detected, one at 0.008 mg/kg and one of 0.006 mg/kg; all others were below the default detection limit. The MPI published MRL for moxidectin in muscle is 0.02 mg/kg [17].

A recent study did not achieve this residue result [18]. In that study a single aberrant levamisole residue exceeding the MRL was reported in fat from nine rising one-year-old wapiti hybrid deer. These deer had been treated with moxidectin injection (0.2 mg/kg) plus oral oxfendazole (18.12 mg/kg) and oral levamisole (15 mg/kg). This combination is a triple action drench recommended to farmers and veterinarians in the deer industry in an attempt to manage the widespread drench resistance [19]. This recommendation was further supported by three slaughter studies [4, 6, 7]. While the Ex/Ox combination is an alternative it is possible that it may not be as effective in controlling resistant* Ostertagia*-type infections as the combination described above particularly in wapiti type deer. Efficacy is undoubtedly more important than WHP as in longer term the deer industry needs effective anthelmintics to succeed. More trial work is needed comparing efficacy of the Ex/Ox combination to the moxidectin injection (0.2 mg/kg) plus oral oxfendazole (18.12 mg/kg) and oral levamisole (15 mg/kg) recommended by Lawrence.

This residue investigation was not undertaken to make any claim about having the WHP recognised by the New Zealand Food Safety Authority. The data obtained from this clinical investigation only adds a small amount to the information that veterinarians have when prescribing the “off label” use of triple combination anthelmintics to their deer farmer clients. The reality is, though, that currently the only products registered for use in deer are known to be widely ineffective and by inference their continuing use is more rapidly selecting for further resistance. “Off label” anthelmintic recommendations are, therefore, essential for many deer farms in New Zealand.

The stability study showed that the Ex/Ox combination was stable for at least six weeks when stored at room temperature or 2–8°C. The sample assay showed anthelmintic content above the expected concentration by 8, 0.4, and 5% for moxidectin, oxfendazole, and levamisole, respectively. This degree of variation is within the allowable tolerances for manufacturing overage and analytical variation for these types of products (J Hurst pers. com.).

In conclusion, a 50/50 mixture of Exodus Pour-On and Oxfen C Plus combination given orally at 1 ml/5 kg bodyweight to weaner red deer was effective against the* Ostertagia*-type parasites on a Taihape deer farm. A 39-day WHP has been shown to be adequate in a single clinical investigation and the mixture was stable for at least six weeks. The novel application of this anthelmintic mixture would appear to have practical application for the New Zealand deer industry, but its use is limited to veterinary prescription.

## Figures and Tables

**Table 1:** Tag numbers and weights for weaner mixed sex red deer with the volume of Exodus Pour-On and Oxfen C Plus 50/50 mixture used per os for treated individuals.

Untreated		Treated		
Tag number	Weight (kg)	Tag number	Weight (kg)	Dose of mix (ml)
322	39.5	305	35.0	7.0
356	36.0	401	26.0	5.5
535	27.5	474	38.0	7.5
583	43.0	713	27.0	5.5
712	34.0	755	45.0	9.0
803	43.0	8944	46.5	9.5

Average weight	37.2		36.3	

**Table 2:** Abomasal worm burdens from weaner mixed sex red deer individually identified into untreated and treated groups. The latter group was dosed per os with Exodus Pour-On Oxfen C Plus 50/50 mixture at 1 ml/5 kg bodyweight 11 days prior to slaughter.

Tag number	*Haemonchus*	*Ostertagia*	*T. axei*
Untreated group			
322	0	1100	500
356	0	1900	700
535	0	2200	100
583	0	1200	100
712	0	4800	100
803	0	4200	30
AM	0	2,566.7	300
GM	0	2,193.2	217.5

Treated group			
305	0	0	0
401	0	0	0
474	0	0	0
713	0	0	0
755	0	0	0
844	0	0	0
AM	0	0	0
GM	0	0	0
%Red AM		>99.9	>99.9
%Red GM		>99.9	>99.9
*P* value^*∗*^		0.0000	0.0000

%Red AM = percentage reduction of arithmetic mean; %Red GM = percentage reduction of geometric mean. ^*∗*^Compared to untreated based on ANOVA using log transformed (count + 1) values.

**Table 3:** Liver selenium concentrations from weaner mixed sex red deer individually identified into untreated and treated groups. The latter group was dosed per os with Exodus Pour-On and Oxfen C Plus 50/50 mixture at 1 ml/5 kg.

Tag number	Liver selenium nmol/kg
Untreated	
322	800
356	580
535	300
583	570
712	430
803	330

Mean	502

Treated	
305	1340
401	2260
474	1880
713	2040
755	2460
844	2290

Mean	2045

(Adequate range 850–5000 nmol/kg).

**Table 4:** Results from laboratory analysis of tissues taken from six rising one-year-old mixed sex red deer dosed per os with a 50/50 mixture of Exodus Pour-On and Oxfen C Plus at a minimum of 1 ml/5 kg 39 days prior to slaughter and two rising one-year-old red deer that were controls.

Deer #	Group	Moxidectin mg/kg	
		Fat	Muscle
1	Treated	0.07	0.008
2	Treated	0.012	<0.005
3	Treated	0.022	<0.005
4	Treated	<0.005	<0.005
5	Treated	0.044	0.006
6	Treated	0.042	<0.005
7	Control	0.007	<0.005
8	Control	<0.005	<0.005

## Abbreviations

DSP: Deer slaughter plant
Ex/Ox: Exodus Pour-On and Oxfen C Plus 50/50 mixture
FEC: Faecal worm egg count
FECRT: Faecal egg count reduction test
G/I: Gastrointestinal
ML: Macrocyclic lactone
MPI: Ministry of Primary Industries
MRL: Maximum residue limit
WHP: Withholding period (for meat).
